# New Layered Polythiophene-Silica Composite Through the Self-Assembly and Polymerization of Thiophene-Based Silylated Molecular Precursors

**DOI:** 10.3390/molecules23102510

**Published:** 2018-09-30

**Authors:** Marie-José Zacca, Danielle Laurencin, Sébastien Richeter, Sébastien Clément, Ahmad Mehdi

**Affiliations:** Institut Charles Gerhardt, UMR 5253-Université de Montpellier, CNRS, ENSCM-CC1701, Place Eugène Bataillon, F-34095 Montpellier CEDEX 05, France; marie-jose.fadous@hotmail.com (M.-J.Z.); danielle.laurencin@umontpellier.fr (D.L.); sebastien.richeter@umontpellier.fr (S.R.)

**Keywords:** self-assembly, lamellar, polythiophene, silica, sol-gel, hybrid material

## Abstract

A new layered hybrid polythiophene-silica material was obtained directly by hydrolysis and polycondensation (sol-gel) of a silylated-thiophene bifunctional precursor, and its subsequent oxidative polymerization by FeCl_3_. This precursor was judiciously designed to guarantee its self-assembly and the formation of a lamellar polymer-silica structure, exploiting the cooperative effect between the hydrogen bonding interactions, originating from the ureido groups and the π-stacking interactions between the thiophene units. The lamellar structure of the polythiophene-silica composite was confirmed by X-ray powder diffraction (XRD) and transmission electron microscopy (TEM) analyses. The solid-state nuclear magnetic resonance (NMR), UV-Vis, and photoluminescence spectra unambiguously indicate the incorporation of polythiophene into the silica matrix. Our work demonstrates that using a polymerizable silylated-thiophene precursor is an efficient approach towards the formation of nanostructured conjugated polymer-based hybrid materials.

## 1. Introduction

Conjugated polymers (CPs) have demonstrated a great potential as an active component in a wide range of electronic and optoelectronic devices, such as polymer solar cells [1,2,3], organic field-effect transistors [4,5,6], polymer light emitting diodes [7,8], organic batteries [9,10], and biosensors [11,12]. Such devices generally involve complex multi-layered architectures, where the synergistic combination of the CPs and the built-in components determine the intrinsic performance of the devices [13,14]. Indeed, the performances of the devices are intimately related to the interfacial contact between the active layers and the nanoscale morphology of the polymer (which is intrinsically linked to the optoelectronic properties of the polymer) [15,16,17]. In this respect, controlling the inter-chain interactions, the orientation, and the conformation of the polymer chains are required to minimize the undesirable charge transfer, increase the charge carrier mobilities, and thus, develop devices with enhanced optical and electronic properties [18,19,20]. CPs are also known to be sensitive to photoinduced oxidation, which affects their electronic and photoconductive properties [21,22].

Incorporation of CPs into an inorganic matrix represents an elegant approach to indirectly control the alignment of polymer chains, to reduce interchain effects by separating the chains and to improve the environmental stability of polymers [23,24,25]. Consequently, enhanced photoluminescence properties have been achieved through the confinement of polymer chains into nanoporous inorganic structures [26,27,28,29]. A critical issue in the conjugated organic-inorganic hybrid and composite materials lie in the inherent incompatibility between the hydrophilic surface of the metal oxide and the hydrophobic nature of the conjugated polymers, which leads to the aggregation of the conjugated chains, a phenomenon often detrimental to the optical properties [30,31,32]. To prevent phase separation, intermolecular non-covalent interactions (e.g., hydrogen and ionic bonding, π-π stacking, hydrophobic) and covalent grafting can be exploited and represent diverse options at the chemist′s disposal [33].

To incorporate CPs into a silica matrix, two approaches are generally used. The first is based on the incorporation of appropriately-functionalized CP into silica by exploiting physical interactions, such as hydrogen bonding [26,30], and aromatic [34] and ionic interactions [27,35,36,37,38]. In this regard, conjugated polyelectrolytes have been widely used to take advantage of the strong ionic interactions operating between the ionic lateral chains and silanol groups. Homogeneous polythiophene/silica composite materials were also successfully prepared through the use of a poly(3-hexylthiophene) copolymer containing hydroxyl groups, due to the strong hydrogen bonding interactions with silanols [30]. More recently, covalently-linked polyfluorene-based organic-inorganic composite and hybrid materials have been reported [39,40]. By controlling either the relative amount of the silyl-functionalized monomer [39] or the degree of branching with ureasil-based silica network [40], the extent of the packing of polyfluorene chains and thus, their conformation can be controlled, allowing the promotion of the higher energy β-phase conformation.

The second approach implies in situ polymerization. Here, the simplest route consists of filling silica pores with monomers and then polymerizing them. Polyaniline, polyalkyne, polythiophene, and poly(phenylene butadiynylene) have been prepared within mesoporous silica, by exploiting this strategy [41,42,43,44]. However, this approach does not guarantee a uniform pore-filling by CPs. More recently, amphiphilic molecules containing polymerizable units were designed, which serve both as a structure-directing agent, operating between the silica precursors, and the polar head groups and monomers [23,45,46,47,48,49,50], through the physical interactions (ionic interactions, hydrogen bonding). The in situ polymerization of the monomers leads to conjugated polymer chains that are uniformly distributed in the inorganic matrix. By judiciously tailoring the size of the alkyl tails or the polar head groups of the monomer-containing surfactants, the mesoscopic order of the CP/silica composite material was selectively tuned [23,45,46,47].

Herein, we have described the preparation of a polythiophene-silica composite material, through the self-assembly and inorganic-polymerization of a judiciously-designed thiophene-containing silylated precursor, in an acid aqueous solution, and the subsequent organic polymerization of the thiophene units. Indeed, cooperative effects originating from the hydrophobic interactions between the alkyl chains, the π-stacking interactions between the thiophene units, and the hydrogen bonding interactions between the ureido groups allow the self-assembly of silylated-thiophene precursors, and lead to ordered thiophene-silica hybrid nanostructures. In situ polymerization allows for the formation of polythiophene, within a highly ordered, inorganic environment. The formation of polythiophene was attested by the solid-state nuclear magnetic resonance (NMR) and UV-Vis spectroscopies. The absorption and emission spectra of the polythiophene-silica composite in the solid state were very similar to those reported for polythiophene, indicating no significant effect of the silica matrix on the electronic structure of polythiophene [51].

## 2. Results and Discussion

### 2.1. Synthesis of the Silylated-Thiophene Precursor

**Scheme 1:**
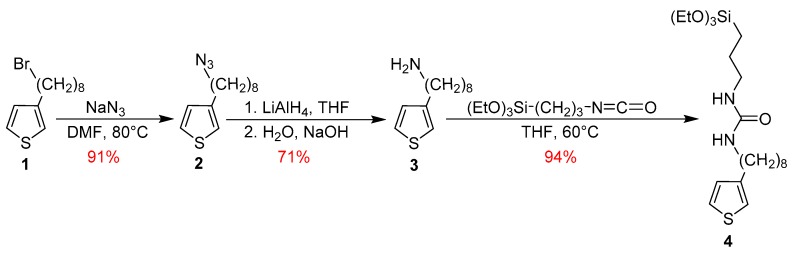
Preparation of the silylated-thiophene precursor **4**.

The monosilylated-thiophene monomer **4** containing a long alkyl side chain and an ureido group was synthesized according to Scheme 1.

3-(8′-Azidooctyl)thiophene (**2**) was first prepared from 3-(8′-bromooctyl)thiophene (**1**), by a nucleophilic aliphatic substitution. The presence of the azido group was evidenced by the azide antisymmetric stretching band at 2092 cm^−1^ in the IR spectrum of **2** (Figure S1 in the Supplementary Materials). The substitution of bromide by azide was also confirmed by ^1^H and ^13^C{^1^H} NMR spectroscopies, with the signals at 3.25 ppm and 51.9 ppm, respectively, corresponding to CH_2_N_3_ (respectively, Figures S2 and S3 in the Supplementary Materials). In the second step, this compound was reduced into the corresponding amine **3** with LiAlH_4_ in THF. The IR spectrum of **3** clearly indicates the presence of the asymmetrical and symmetrical N-H stretching vibrations at 3400 and 3300 cm^−1^, characteristic of the primary amine function (Figure S4 in the Supplementary Materials). ESI-TOF mass spectrometry (positive mode) also revealed the expected *m/z* = 212.1 Da [M + H^+^]. Finally, the reaction between the primary amine **3** and 3-(isocyanatopropyl)triethoxysilane quantitatively afforded the precursor **4**. Formation of **4** was confirmed by ^1^H, ^13^C{^1^H}, and ^29^Si{^1^H} NMR spectroscopies in CDCl_3_ (respectively, Figures S7–S9 in the Supplementary Materials). The presence of the trialkoxysilyl group was attested in the ^1^H-NMR spectrum of **4** by the triplet and quadruplet, at 3.78 and 1.19 ppm, respectively, which were assigned to the OCH_2_ and CH_3_ groups (Figure S7 in the Supplementary Materials). As expected, the ^29^Si{^1^H} spectrum of **4** exhibits only one signal at −45.2 ppm, corresponding to RSi(OEt)_3_ moieties (Figure S9 in the Supplementary Materials).

### 2.2. Synthesis of the Lamellar Thiophene-Silica Hybrid Material

**Scheme 2:**
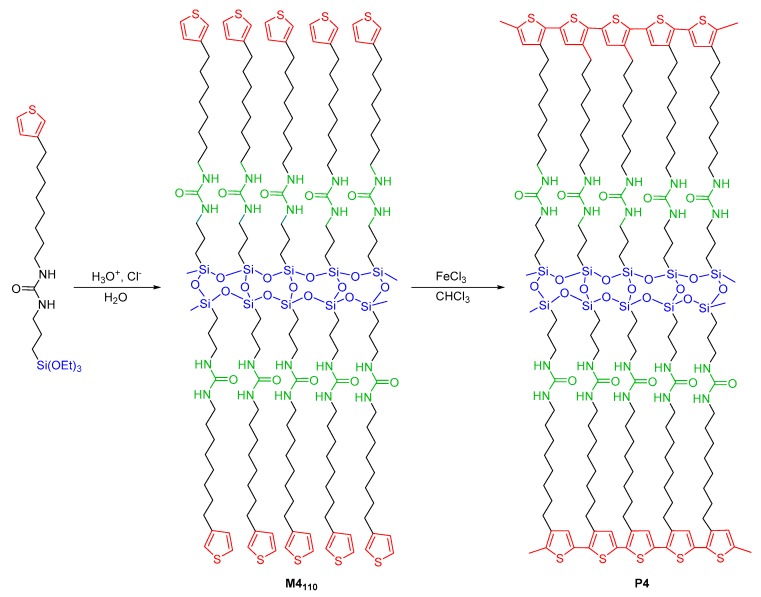
The synthetic route for the preparation of thiophene and polythiophene-based hybrid materials **M4_110_** and **P4**.

The hydrolysis and condensation of the monosilylated precursor **4** in suspension in water at pH = 1.5 were first performed at room temperature for 48 h (Scheme 2). In fact, at this pH, the hydrolysis takes place while the polycondensation is induced by the self-assembly of the precursor to give the hybrid material **M4** [52].

**Figure 1:**
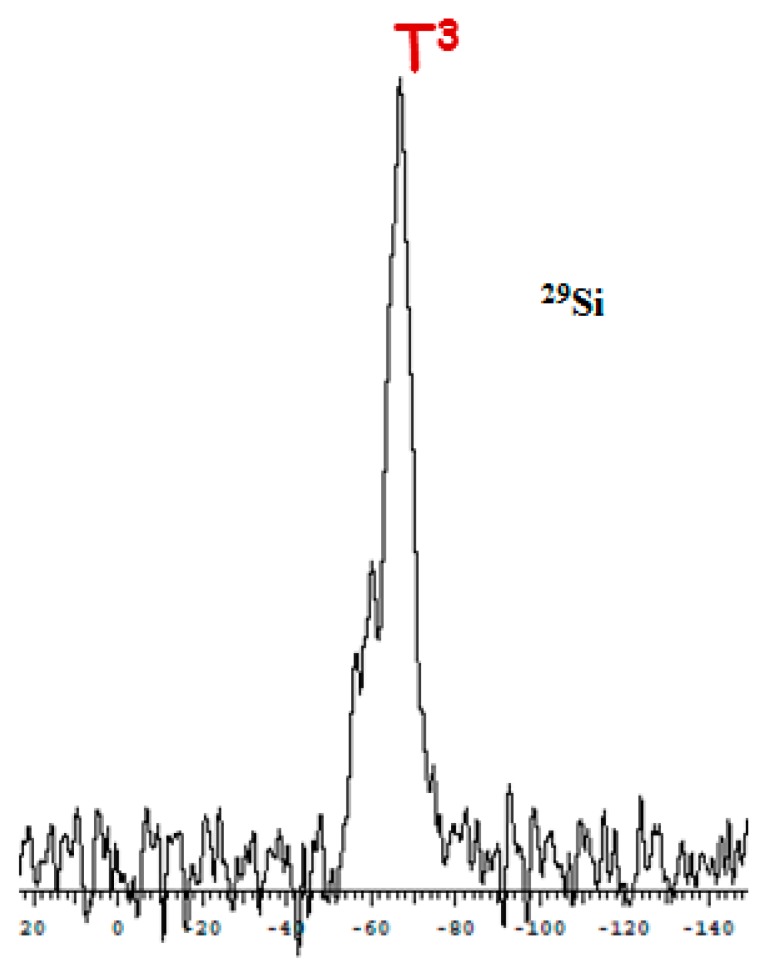
^29^Si CPMAS solid-state NMR spectrum of the hybrid material **M4_110_**.

After this time, the obtained precipitate was filtered, treated by following the usual work-up and dried (see Experimental Section). The IR spectrum of the resulting solid **M4_25_** revealed the presence of a very broad band at 3200 cm^−1^, corresponding to the residual O-H bonds and indicating an incomplete polycondensation reaction (Figure S10 in the Supplementary Materials). To improve the polycondensation rate, the hybrid material **M4_25_** obtained previously was suspended in acidic water (pH = 1.5) at 110 °C for 24 h. Treatment of **M4_25_** in these conditions quantitatively afforded **M4_110_** as a white powder. The ^29^Si CPMAS NMR spectrum of **M4_110_** showed a high rate of T^3^ species (major peak around −66.0 ppm) (Figure 1). The higher degree of polycondensation for **M4_110_** was also confirmed from the results of the elemental analyses since values of C, H, N, and S content closer to theoretical ones were found.

The extent of the intermolecular hydrogen bonding due to the ureido moieties was examined in **M4_110_** by FT-IR spectroscopy. Here, the differences (∆ν) between the two vibration amide modes (amide I (ν_C=O_) and amide II (δ_NH_)) of the ureido group were used as a probe to evaluate the strength of the hydrogen bonds between the ureido groups: the lower the ∆ν values, the stronger the hydrogen bonding interactions [53,54,55]. From the IR spectrum of **M4_110_**, a ∆ν of 50 cm^−1^ was calculated. This difference was of the same magnitude as the ones found for the monosilylated precursors of the formula (EtO)_3_Si(CH_2_)_3_NH(C=O)NHC_n_H_2n+1_ (*n* = 8 and 12), and indicated significant hydrogen-bonding interactions (see Experimental section and Figure S8 in the Supplementary Materials) [55]. It was also interesting to note that no band was observed at 1680 cm^−1^ indicating no free ureido groups in this hybrid material.

**Figure 2:**
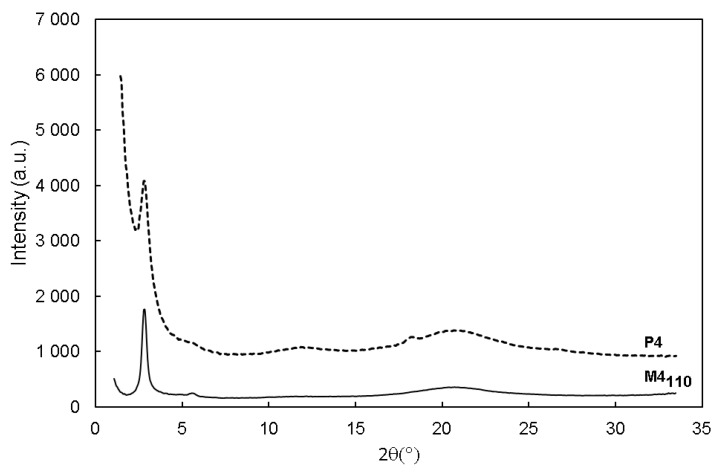
X-ray powder diffraction (XRD) diffractograms of thiophene-and-polythiophene-based hybrid materials **M4_110_** and **P4**.

**Figure 3:**
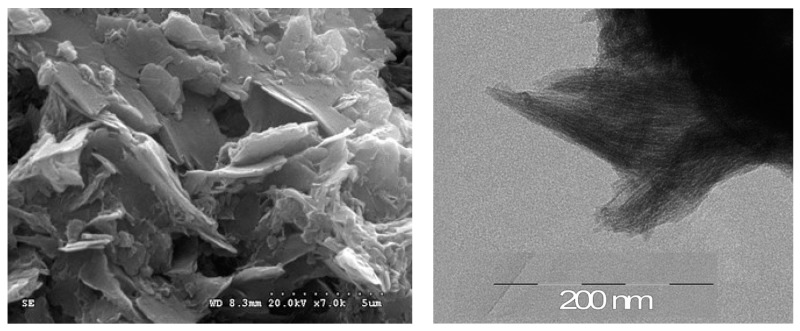
SEM (**left**) and TEM (**right**) images of the lamellar structure of **M4_110_**.

The X-ray powder diffraction (XRD) pattern of **M4_110_** exhibited diffraction peaks, which are characteristic of a lamellar structure (Figure 2). Indeed, peaks at 2θ ~ 2.86° and 5.57° were observed, corresponding to the (100) and (200) orders with an interlayer distance of 3.1 nm (calculated using Bragg’s law nλ = 2d × sinθ with λ(CuK_α1_) = 1.542 Å). This value was different from the theoretical distance estimated by the ChemDraw3D calculation (3.5 nm) and corresponding to the two *N*-propyl-*N*′-(3-octylthiophene)urea units. This difference could be explained by the inclination of the alkylene chains with respect to the plane formed by the condensed siloxane units, which was not taken into account for the ChemDraw 3D calculation [56]. It is worth noting that a broad reflection, centered at 2 θ ~ 21° was also observed, corresponding to a distance of 4.2 Å, which is a typical value for thiophene interchain distances and also close to what can be found among siloxane units [56,57]. To determine the morphology of the hybrid material **M4_110_** on a microscopic scale, SEM analyses were performed. SEM images revealed that **M4_110_** exhibited a lamellar morphology (Figure 3, left). Further evidence for the lamellar structure was given by transmission electron microscopy (TEM) (see Figure 3, right), which displayed defined striped patterns. The thermal fragility of the material, due to a high organic content, explains the low-image resolution.

Taken together, all these data indicate that the self-assembly of the hybrid materials operated as expected, through the formation of hydrogen bonding interactions between the ureido groups, van der Waals interactions between the alkylene chains, and π-π stacking interactions between thiophene units [58]. As such, the acid hydrolysis of alkoxysilyl groups into silanol in these “*pre-organized*” molecular structures allowed obtaining ordered hybrid materials.

### 2.3. The preparation of Lamellar Polythiophene-Silica Hybrid Material

The dense packing of the thiophene groups in the interlamellar space of the hybrid materials allowed their polymerization to form the lamellar polythiophene-silica hybrid material **P4** (Scheme 2). In situ thiophene polymerization was carried out by mixing **M4_110_** with anhydrous FeCl_3_, a well-known thiophene polymerization oxidant [59], in chloroform, and stirring the mixture, at room temperature, for 72 h. The color of the reaction medium gradually shifted from orange (beginning of the reaction) to dark green, after 72 h (Figure S11 in the Supplementary Materials), due to the change in oxidation state of iron (III) to iron (II). After three days, the suspension was filtered and the resulting powder was thoroughly washed with water and methanol to eliminate the residual iron salts.

The hybrid material **P4** was analyzed by ^13^C solid-state NMR, and compared to **M4_110_** (Figure S12 in the Supplementary Materials). After polymerization, noticeable changes were observed in the ^13^C chemical shifts of both the aromatic and alkyl carbons, showing that their local environment was modified. Moreover, a significant broadening of the ^13^C resonances was observed, especially for the CH_2_ groups, which could be explained by a decrease in “mobility” of the alkyl chains, due to the rigidification of the material. Although the detailed assignment of the ^13^C resonances has not been achieved so far (as it would require additional high-resolution solid-state NMR experiments), these observations strongly suggest that some interconnection between the thiophene rings had occurred. Moreover, the XRD pattern of **P4** (Figure 1) shows the preservation of the diffraction peaks, after polymerization, indicating that the lamellar structure was not affected by this polymerization process. Two additional peaks at 2θ = 11.84° and 18.20°, corresponding to d-spacings of 7.5 Å and 4.8 Å, respectively were noticed. These peaks might correspond to the higher-order reflection peaks of the obtained polymer [60,61]. The diffraction peak at 2θ ~ 21° might also indicate the persistence of the interactions between the chains in the polymer backbones [62,63]. Thermal stability of **P4** was evaluated by the thermal gravimetric analysis (TGA), from 25 °C to 600 °C, in air. The obtained curve (Figure S13 in the Supplementary Materials) showed that the hybrid material remained stable up to 180 °C and the loss of mass percentage between 180 °C and 600 °C was estimated to be around 75%, which is close to the theoretical percentage (78%).

**Figure 4:**
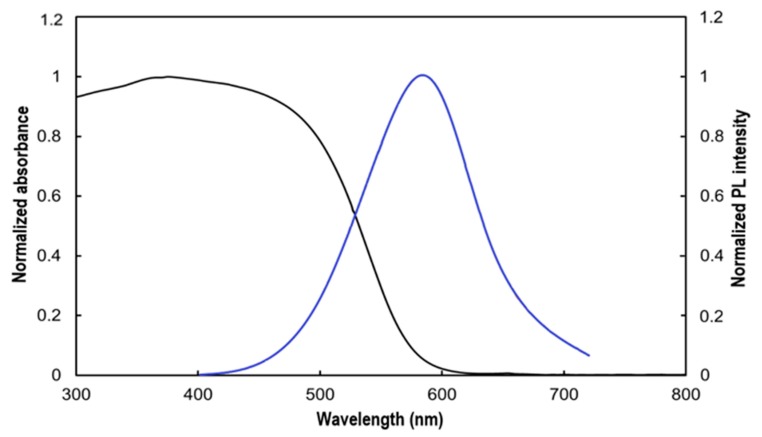
UV-vis absorption (black line) and fluorescence (blue line, λ_exc_ = 370 nm) spectra of the lamellar polythiophene-silica hybrid material **P4** in the solid state.

In situ polymerization leads to a change in color from white for **M4_110_** to a reddish orange for **P4**, suggesting the formation of conjugated polythiophene chains. UV-vis absorption and fluorescence spectra were recorded to investigate the optical properties of the resulting polythiophene-silica hybrid material **P4** (Figure 4). **P4** exhibited the maximum absorption and emission wavelengths, at 385 and 582 nm, respectively. Theoretical calculations and experimental measurements have shown that a linear relationship was typically observed between the adiabatic transition energy (E_00_) and the inverse of the number of thiophene rings, allowing the determination of the effective conjugation length [64,65]. E_00_ was determined experimentally, by determining the intersection of the normalized absorption and emission spectra. Thus, by exploiting the relationship between E_00_ and the inverse of the number of thiophene units, the effective conjugation length (ECL) could be estimated. An ECL of ~2.9 nm, corresponding to seven substituted thiophene rings was found from the intersection of the absorption and emission spectra, at 527 nm. The length of alkyl chains at the third position of the thiophene ring, in the polymerization of the thiophene monomers, has been found to significantly influence the effective conjugation length. Indeed, long alkyl chains are more flexible and accommodating, such as dodecyl chains, and lead to a larger effective conjugation length than the shorter ones [66,67]. As such, the long alkyl chains at the third position of the thiophene ring, in **M4_110_** must have favoured effective polymerization, and thus, the conjugation length observed. This value also compares well with those previously obtained by Wang et al. for mesostructured poly(3-dodecylthiophene)-silica composite particles [46].

## 3. Experimental Method

### 3.1. Instrumentation and Methods

Reactions were performed under argon, using oven-dried glassware and Schlenk techniques. 3-(8′-bromooctyl)thiophene was prepared according to the literature procedure [68]. Sodium azide (99.5%), LiAlH_4_ (1.0 M in THF), 3-(triethoxysilyl)propyl isocyanate (95%), anhydrous iron chloride (III) (97%) were purchased from Sigma-Aldrich (Saint Louis, MO, USA). Dry DMF and THF were obtained by using a solvent purification system, PureSolve MD5 purchased from Inert Technology (Amesbury, MA, USA). Preparative purifications were performed by silica gel flash column chromatography (Merck^®^ 40–60 µM, Darmstadt, Germany). Solvents used as eluents were technical grade. IR spectra were recorded on a Perkin Elmer Spectrum 2 FTIR spectrophotometer (Perkin Elmer, Waltham, MA, USA). ^1^H, ^13^C{^1^H} were recorded with a Bruker Avance 600 spectrometer (^1^H 600.26 MHz, ^13^C{^1^H} 150.96 MHz) (Bruker, Billerica, MA, USA). ^29^Si{^1^H} was acquired on a Bruker Avance 400 spectrometer (79.46 MHz) (Bruker, Billerica, MA, USA). Mass spectra (MS) were recorded on ESI-TOF Q instruments (Waters, Milford, MA, USA) in the positive mode. Elemental analyses were performed on a Vario MICRO CHNS elemental analyzer (Elementar, LangenselboldGermany). Powder X-ray diffraction experiments were carried out on a high-resolution Bonse-Hart camera with two germanium channel cuts for very small q values (Malvern Panalytical, Worcestershire, United Kingdom). The wavelength used was 1.542 Å (CuK_α_ radiation). Scanning Electron Microscopy (SEM) images were obtained with a Hitachi S2600N microscope (Hitachi, Tokyo, Japan). Transmission Electron Microscopy (TEM) observations were carried out at 100 kV (JEOL 1200 EXII) (JEOL, Tokyo, Japan). Samples for TEM measurements were prepared by embedding the hybrid material in AGAR 100 resin, followed by ultramicrotomy techniques and deposition on copper grids. The solid-state NMR spectrum for ^29^Si was recorded on a Varian VNMRS 300 Solid spectrometer (Varian, Palo Alto, CA, USA), at a magnetic field strength of 7.05 T. A 7.5 mm MAS probe was used with a spinning rate of 5 kHz. Single pulse experiments with a continuous wave ^1^H decoupling were used for ^29^Si NMR, with 2 μs π/2 pulse duration and a recycle delay of 60 s. A recycle delay of 200 s was found necessary to allow a full ^29^Si relaxation, although this did not lead to any change in the relative ratio of the individual components on the spectral decomposition. Thus, data with a recycle delay of 60 s could be considered as quantitative. ^13^C solid-state NMR analyses were performed on a Varian VNMRS 600 MHz NMR spectrometer (B_0_ = 14.1 T, ν_0_(^1^H) = 599.8MHz and ν_0_(^13^C) = 150.8MHz). Experiments were performed using a Varian T3 HXY 3.2 mm probe, tuned to ^1^H and ^13^C, and spinning at 18 kHz. A ^1^H-^13^C CPMAS (Cross Polarization Magic Angle Spinning) pulse sequence was used, with a ^1^H 90° excitation pulse of 2.5 μs, followed by a ramped contact pulse of 2 ms. Spinal-64 ^1^H decoupling was applied during acquisition (100 kHz RF). The recycle delay was set to 1–2 s, depending on the sample, and the number of transients acquired ranged from 1430 to 7670. The ^13^C chemical shifts were referenced to TMS (tetramethylsilane), using the deshielded resonance of adamantane (38.5 ppm) as a secondary reference.

### 3.2. Synthesis of 3-(8′-Azidooctyl)thiophene *(**2**)*

To a solution of 3-(6′-bromooctyl)thiophene (**1**) (6.34 g, 23.0 mmol) in DMF (100 mL) was added to sodium azide (3 g, 34.5 mmol, 1.5 eq). The mixture was stirred at 80 °C, overnight. After cooling, the mixture was diluted with CHCl_3_ (75 mL) and washed three times with water (3 × 50 mL). The organic layer was dried over MgSO_4_ and the organic solvent was removed under reduced pressure. Compound **2** was obtained as a colorless liquid (4.98 g, 91%). IR (ATR): 2092 cm^−1^ (νN_3_). ^1^H-NMR (CDCl_3_): 7.23 (m, 1 H, H_ar_), 6.93 (m, 2 H, 1 H_ar_), 3.25 (t, 2 H, CH_2_N_3_, ^3^*J*_H-H_ = 6.8 Hz), 2.63 (t, 2H, CH_2_Thio, ^3^*J*_H-H_ = 7.1 Hz), and 1.72–1.19 (m, 12H, CH_2_). ^13^C{^1^H} NMR (CDCl_3_): δ = 143.7, 128.7, 125.5, 120.2 (C_ar_), 51.9 (CH_2_-N_3_), 30.9, 30.7, 29.7, 29.6, 29.2, and 27.1 (CH_2_) ppm. ESI-MS: cacld. for [C_12_H_20_NS]^+^ 210.1 [M + H-N_2_], was found to be 210.1.

### 3.3. Synthesis of 3-(8′-Aminooctyl)thiophene *(**3**)*

In a 250 mL two-neck round-bottom flask equipped with a refrigerant, **2** (4.74 g, 20.0 mmol) was dissolved in THF (40 mL). After cooling to 0 °C, LiAlH_4_ (40 mL, 40.0 mmol, 1.0 M solution in THF) was added in drops. After the reaction was judged complete by TLC (eluent: *n*-pentane/CH_2_Cl_2_ 1/1), the reaction was quenched with 15% aqueous NaOH (1.6 mL), then followed by ice water (3 × 1.6 mL). The precipitated aluminum salts were filtered. The filtrate was dried with anhydrous MgSO_4_ and the solvent was removed by evaporation. Compound **3** was obtained as a colorless liquid (4.98 g, 71 %). IR (ATR): 3400, 3300 cm^−1^ (νNH_2_). ^1^H-NMR (CDCl_3_): δ = 7.23 ppm (s, 1H, Th), 6.93 (m, 2H, Th), 2.68 (t, 2H, CH_2_-Th, ^3^*J*_H-H_ = 7.5 Hz), 2.62 (t, 2H, CH_2_-NH_2_, ^3^*J*_H-H_ = 6.7 Hz), and 1.74–1,15 (m, 12H, CH_2_) ppm. ^13^C{^1^H} NMR (CDCl_3_): δ = 143.0, 128.7, 125.5, 120.2 (Th), 51.9 (CH_2_-N), 30.9, 30.6, 29.7, 29.6, 29.5, 29.2, and 27.1 (CH_2_) ppm. ESI-MS: cacld. for [C_12_H_22_NS]^+^ 212.1 [M + H], was found to be 212.1.

### 3.4. Synthesis of the Thiophene Silylated Precursor *(**4**)*

To a solution of **3** (2.89 g, 14.0 mmol) in THF (35 mL), 3-isocyanatopropyltriethoxysilane (3.8 mL, 15.0 mmol) was slowly added under stirring and the reaction was heated, at 60 °C for 2 h. THF was evaporated under reduced pressure to obtain a viscous oil and *n*-pentane (50 mL) was then added to precipitate **4**. The liquid supernatant containing the excess of 3-isocyanatopropyltrithoxysilane was removed and the precipitate was washed three more times with *n*-pentane (25 mL). The compound **4** was then dried under vacuum and obtained as a white solid in 94% yield (6.03 g). IR (ATR): 3312 cm^−1^ (νNH), 1628 cm^−1^(νCO_amide_). ^1^H-NMR (CDCl_3_): δ = 7.19 (m, 1H, Th), 6.88 (m, 2H, Th), 5.20–4.86 (m, 2H, NH-CO-NH), 3.78 (q, 6H, O-CH_2_, ^3^*J*_H-H_ = 7.0 Hz), 3.09 (m, 4H, CH_2_-N), 2.58 (t, 2H, CH_2_, ^3^*J*_H-H_ = 7.3 Hz), 1.24–1.68 (m, 14H, CH_2_), 1.19 (t, 9H, CH_3_, ^3^*J*_H-H_ = 7.0 Hz), and 0.60 (t, 2H, CH_2_-Si, ^3^*J*_H-H_ = 8.1 Hz) ppm. ^13^C{^1^H} NMR (CDCl_3_): δ = 158.9 (CO), 143.1, 128.3, 125.1, 119.8 (Th), 58.4, 42.9, 40.5, 30.6, 30.4, 30.3, 29.5, 29.4, 29.3, 27.0, 23.7, 18.3, and 7.8 ppm. ^29^Si{^1^H} NMR (CDCl_3_) δ = −45.2 ppm. Anal. calcd. for C_22_H_42_N_2_O_4_SSi (458.26): %C 57.60, %H 9.23; %N 6.11, %S 6.99 was found to be %C 57.76, %H 9.04; %N 5.98, and %S 6.90.

### 3.5. Synthesis of the Lamellar Thiophene-silica Hybrid Materials

Compound **4** (4.58 g, 10.0 mmol) was placed into a Schlenk tube with acidified water (30 mL, pH = 1.5) and stirred, at room temperature for 48 h. The resulting solid was filtered and dried under vacuum, leading to material M4_25_. Half of this solid was then placed into a Schlenk tube and 30 mL of water, at pH = 1.5 was added. The resulting solution was stirred at 110 °C for 24 h. After cooling, the white precipitate was isolated by filtration, washed three times with water and then dried under vacuum leading to material **M4_110_**.

**M4_25_**: IR (ATR): ν = 3311 (νNH stretching), 3200 (νOH stretching), 2926, 2845 (νCH_2_), 1626 (νC=O stretching), and 1572 (δNH bending) cm^−1^. Anal. calcd. for C_16_H_27_N_2_O_2.5_SSi (347.15): %C 55.29, %H 7.83; %N 8.06, %S 9.22 was found to be %C 40.30, %H 6.97; %N 5.93, and %S 6.60.

**M4_110_**: IR (ATR): ν = 3318 (νNH stretching), 2924, 2852 (νCH_2_), 1622 (νC=O stretching), and 1572 (δNH bending) cm^−1^. Anal. calcd for C_16_H_27_N_2_O_2.5_SSi (347.15): %C 55.29, %H 7.83; %N 8.06, %S 9.22 was found to be %C 53.57, %H 8.97; %N 7.89, and %S 9.16.

### 3.6. In Situ Polymerization of Thiophene Groups

Anhydrous iron chloride (III) (0.8 g) and dried chloroform (10 mL) were placed in a round-bottom flask under inert atmosphere. Thiophene-silica composite material (0.3 g) was added and the resulting mixture was stirred, at room temperature for 72 h. The suspension was then filtered and washed with water (2 × 10 mL) and methanol (2 × 10 mL) by centrifugation, and dried under vacuum leading to an orange-red solid.

## 4. Conclusions

A new layered polythiophene-silica hybrid material was prepared by hydrolysis and polycondensation of a judiciously-designed, monosilylated-thiophene precursor and its subsequent polymerization. By exploiting the self-assembly of this silylated-thiophene precursor, through van der Waals interactions of alkylene chains, π-stacking interactions between the thiophene units, and hydrogen bonding interactions between ureido groups, a lamellar thiophene-silica hybrid material was achieved. Then, the dense packing of thiophene groups in the central region of the lamellar silica structure allowed the in situ polymerization to form an ordered polythiophene-silica composite. The formation of nanostructured polythiophene-silica was confirmed by combining XRD, TEM, solid-state NMR, and UV-Vis absorption spectroscopy. The UV-Vis absorption and emission spectra of the polythiophene-silica composite in the solid state were very similar to those reported for polythiophene, indicating no significant effect of the silica matrix on the electronic structure of polythiophene. We believe that our strategy represents an efficient approach towards the formation of nanostructured, conjugated, polymers-based hybrid materials with tunable properties, through a judicious design of the silylated monomer.

## Supplementary Materials

The following are available online at http://www.mdpi.com/1420-3049/23/10/2510/s1, Figure S1: ATR-FT-IR spectrum of **2**, Figure S2: ^1^H-NMR spectrum of **2** in CDCl_3_, Figure S3: ^13^C{^1^H} NMR spectrum of **2** in CDCl_3_, Figure S4: ATR-FT-IR spectrum of **3**, Figure S5: ^1^H-NMR spectrum of **3** in CDCl_3_, Figure S6: ^13^C{^1^H} NMR spectrum of **3** in CDCl_3_, Figure S7: ^1^H-NMR spectrum of **4** in CDCl_3_, Figure S8: ^13^C{^1^H} NMR spectrum of **4** in CDCl_3_, Figure S9: ^29^Si{^1^H} NMR spectrum of **4** in CDCl_3_, Figure S10: IR spectra of hybrid materials **M4_25_** (bottom) and **M4_110_** (top), Figure S11: Color evolution during the chemical polymerization of thiophene units in M4_110_. Figure S12: ^13^C-CPMAS solid-state NMR spectra of **M4_110_** and **P4**. Figure S13: TGA curve of lamellar polythiophene-silica hybrid material **P4**.
