# Supplementary material for: New Layered Polythiophene-Silica Composite Through the Self-Assembly and Polymerization of Thiophene-Based Silylated Molecular Precursors
Source: Molecules. 2018 Sep 30;23(10):2510. doi: 10.3390/molecules23102510 (PMC6222596; doi:10.3390/molecules23102510)
Supplement: Supplementary file 1 [file molecules-23-02510-s001.pdf]

Supplementary materials for:

Layered polythiophene-silica composite through self-assembly and polymerization of thiophene-based silylated molecular precursors

Marie-José Zacca, Danielle Laurencin, Sébastien Richeter, Sébastien Clément * and Ahmad Mehdi *

Institut Charles Gerhardt, UMR 5253 - Université de Montpellier, CNRS, ENSCM – CC1701, Place Eugène Bataillon, F-34095 Montpellier Cedex 05, France.

<u>Table of contents</u>:

ATR-FT-IR spectrum of **2** **P.2**

^{1}H NMR spectrum of **2** in $CDCl_3$ **P.2**

^{13}C{^{1}H} NMR spectrum of **2** in $CDCl_3$ **P.3**

ATR-FT-IR spectrum of **3** **P.3**

^{1}H NMR spectrum of **3** in $CDCl_3$ **P.4**

^{13}C{^{1}H} NMR spectrum of **3** in $CDCl_3$ **P.4**

^{1}H NMR spectrum of **4** in $CDCl_3$ **P.5**

^{13}C{^{1}H} NMR spectrum of **4** in $CDCl_3$ **P.5**

^{29}Si{^{1}H} NMR spectrum of **4** in $CDCl_3$ **P.6**

ATR-FT-IR spectra of hybrid materials **M4$_{25}$** (bottom) and **M4$_{110}$** (top) **P.6**

Colour evolution during the chemical polymerization of thiophene units in **M4$_{110}$** **P.7**

^{13}C CPMAS solid state NMR spectra of **M4$_{110}$** and **P4** **P.7**

TGA curve of lamellar polythiophene-–silica hybrid material **P4** **P.8**

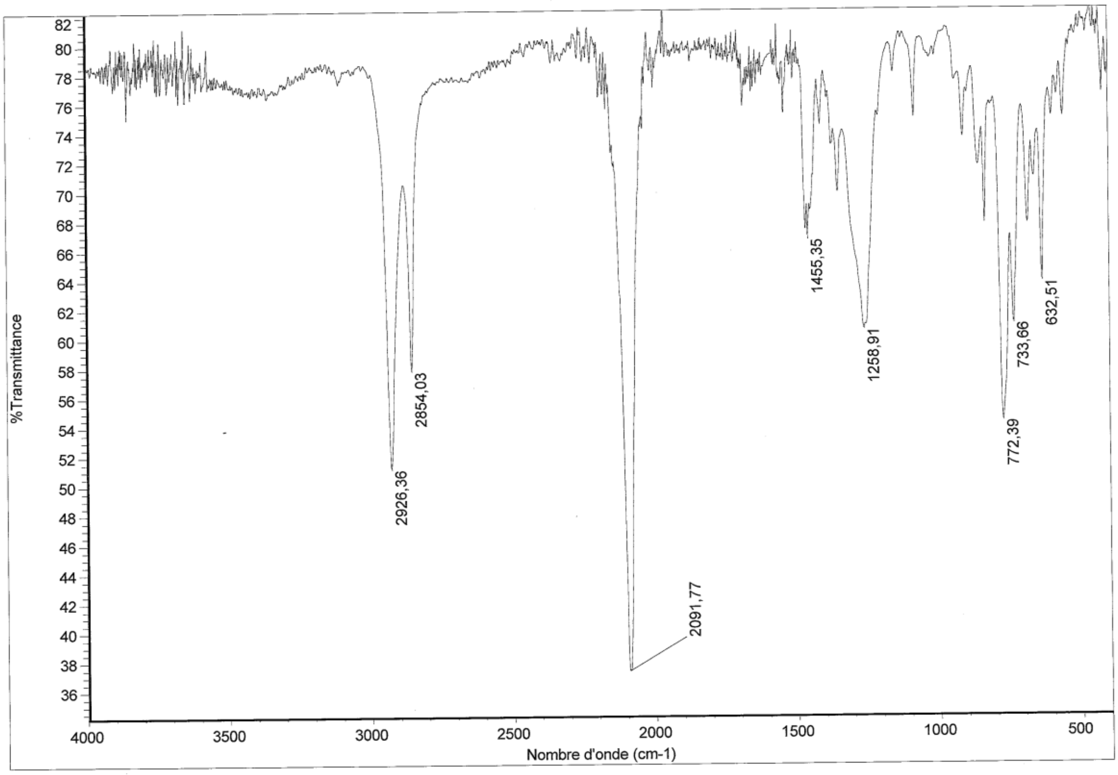

Figure S1 : ATR-FT-IR spectrum of **2**.

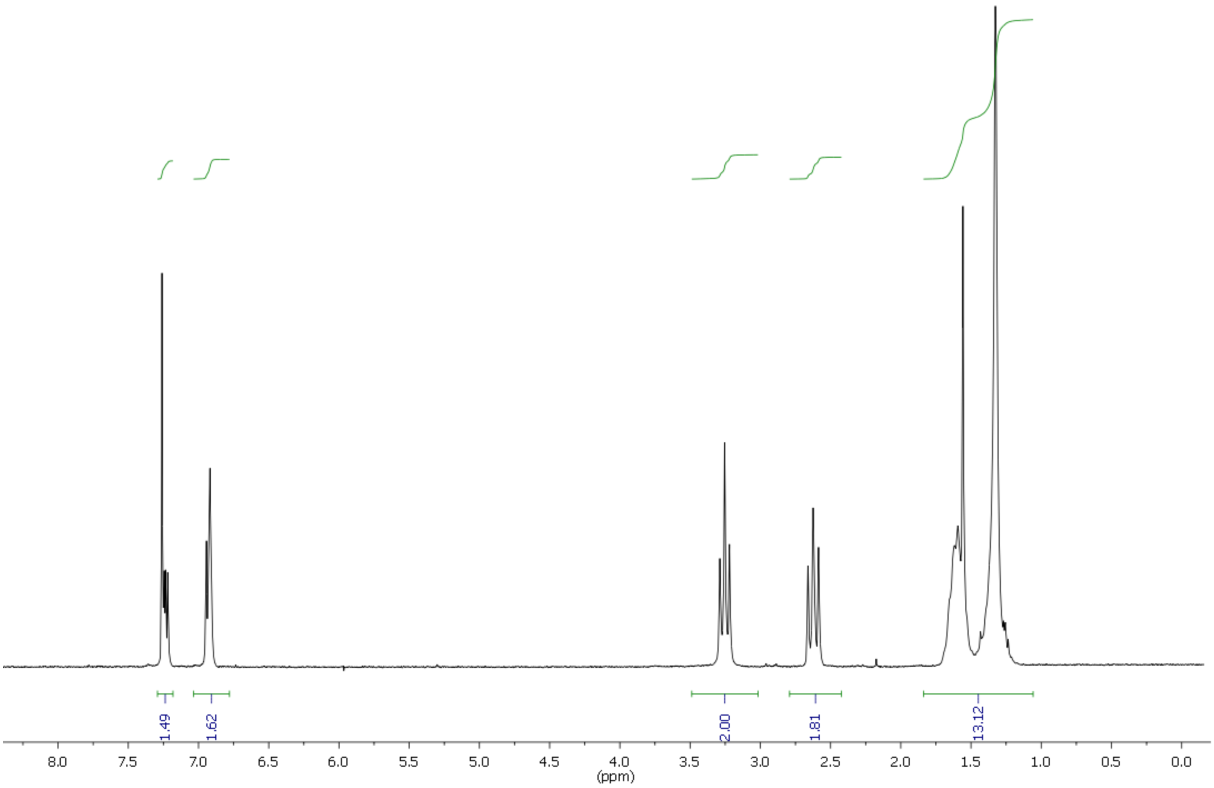

Figure S2 : ^{1}H NMR spectrum of **2** in $CDCl_3$.

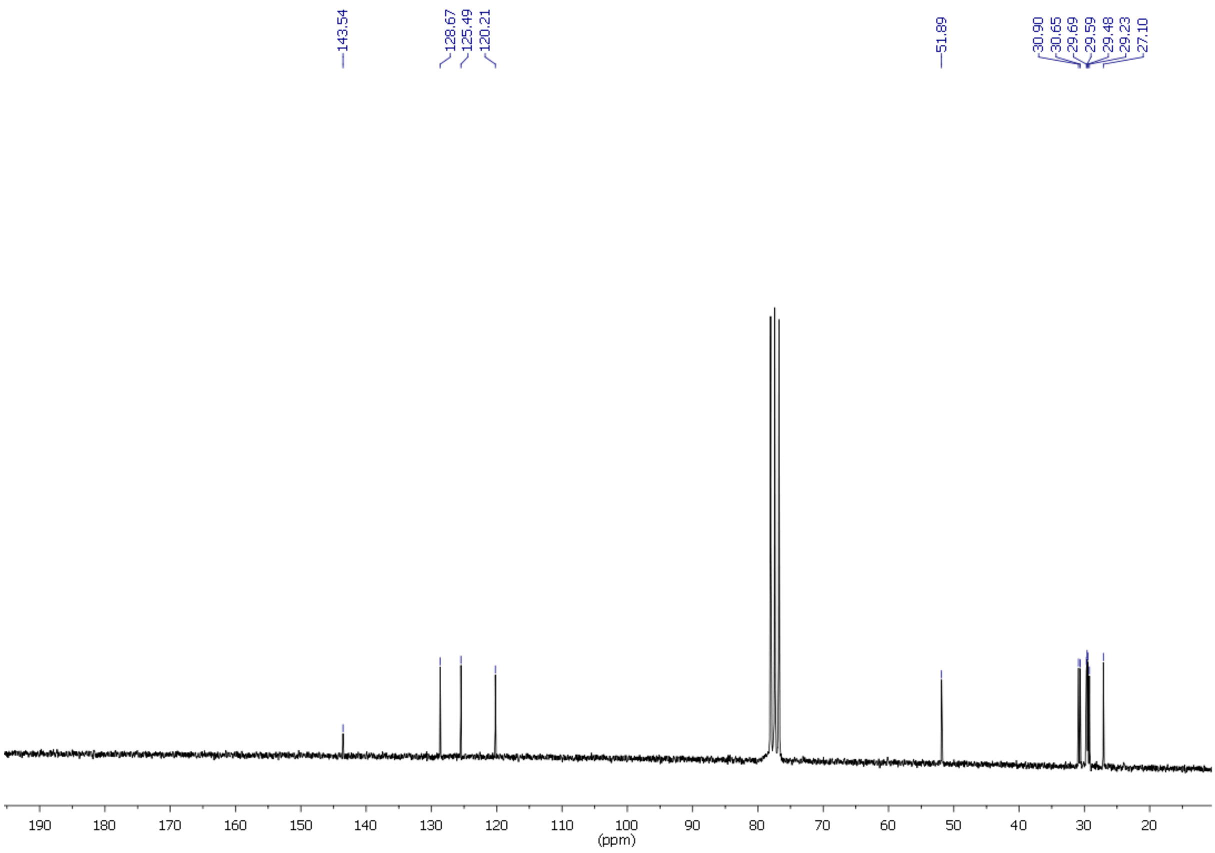

Figure S3 : $^{13}C\{^{1}H\}$ NMR spectrum of **2** in $CDCl_3$.

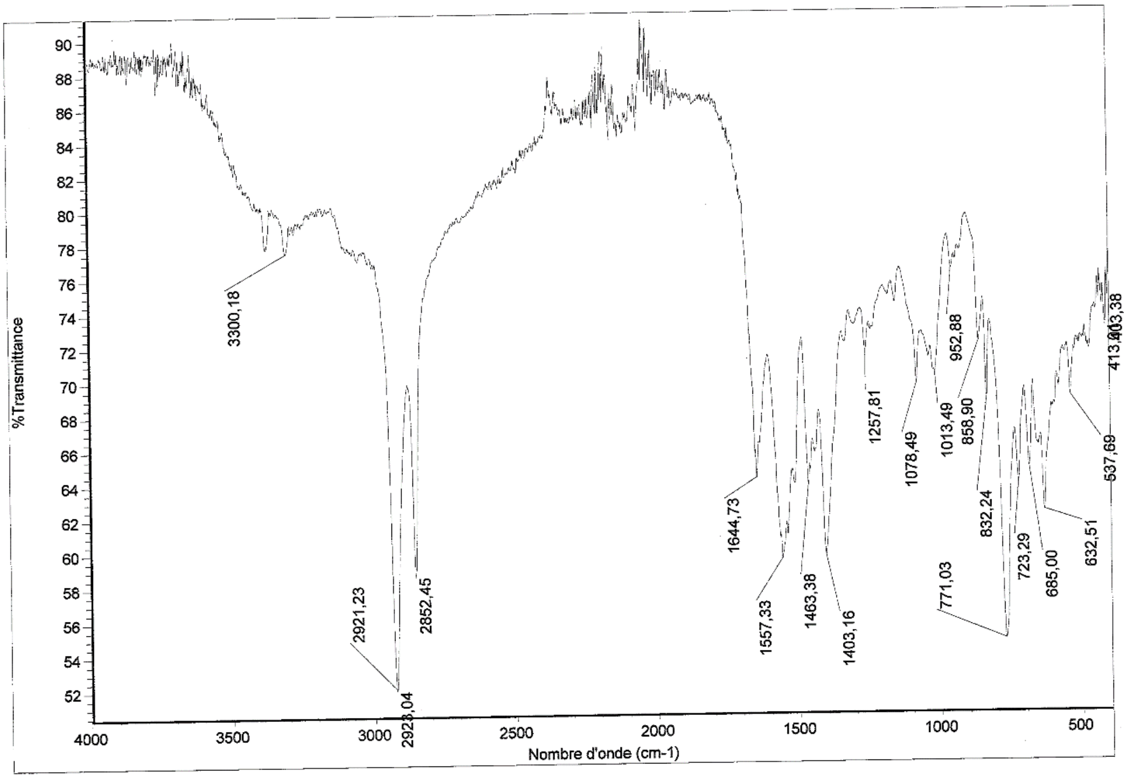

Figure S4: ATR-FT-IR spectrum of **3**.

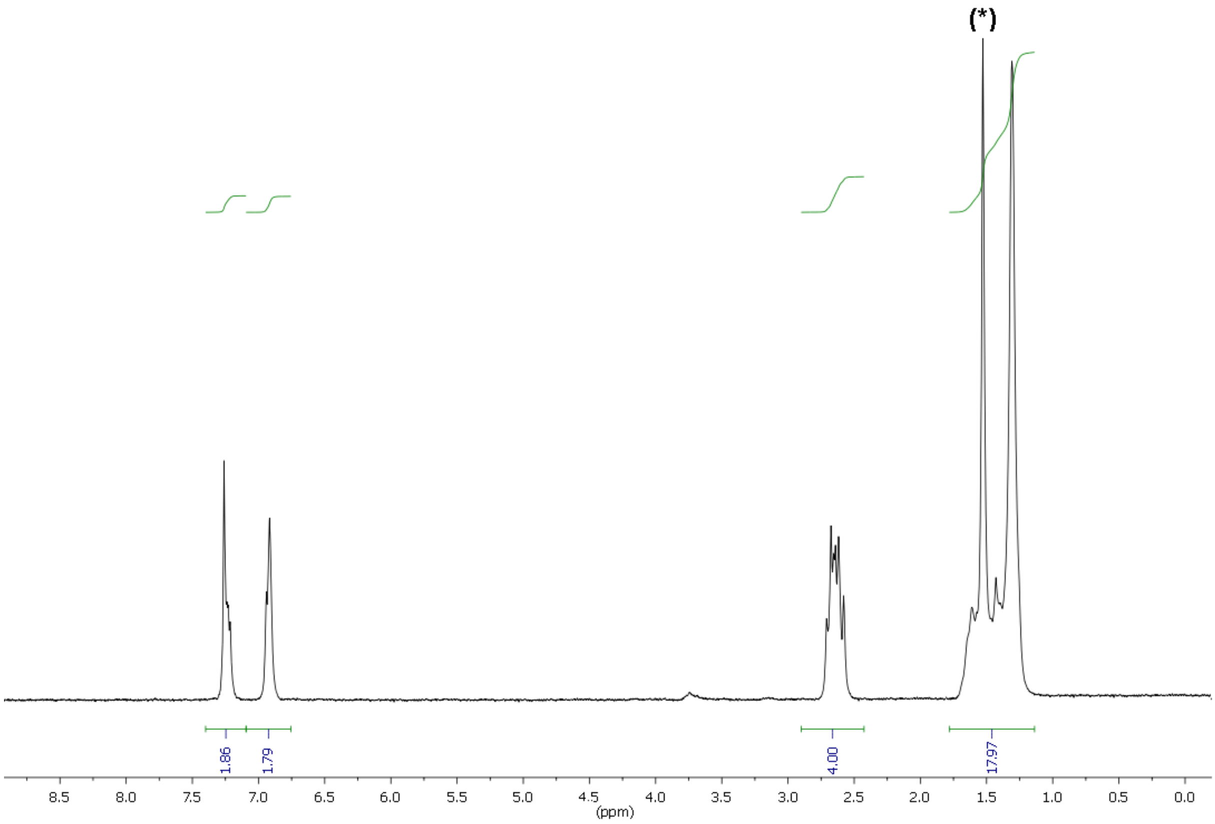

Figure S5 : ^{1}H NMR spectrum of **3** in $CDCl_3$.(*) Water.

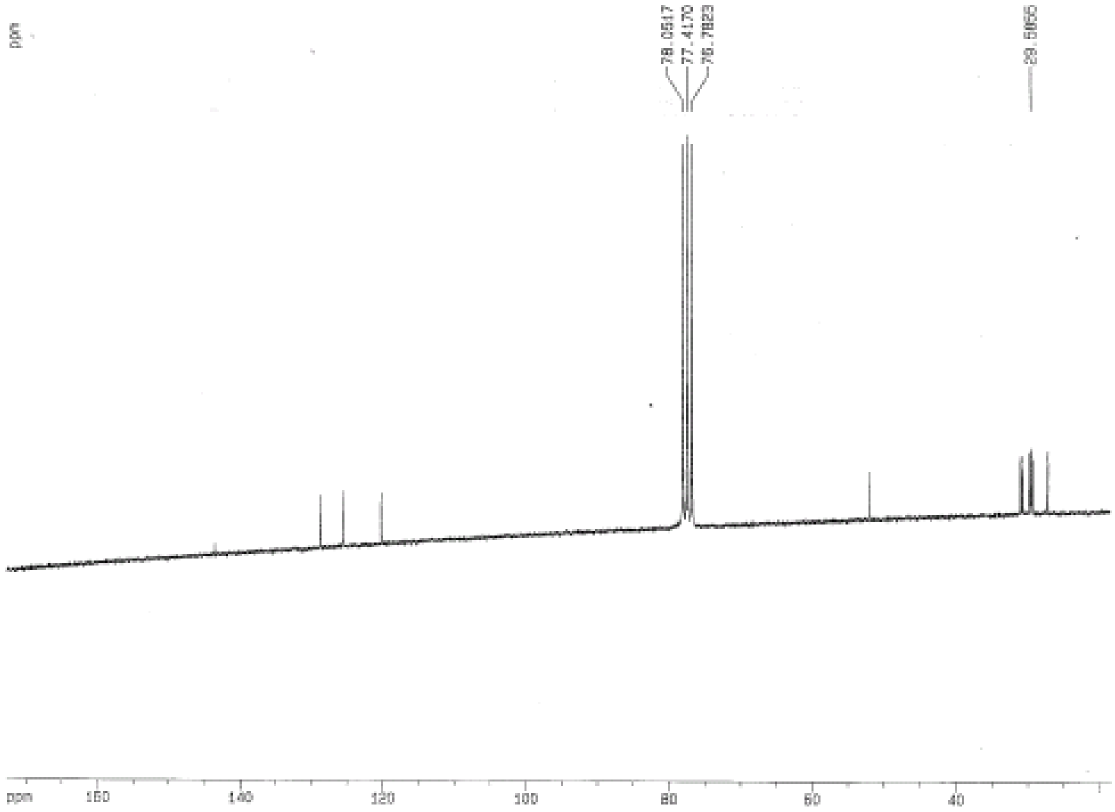

Figure S6 : $^{13}C\{^{1}H\}$ NMR spectrum of **3** in $CDCl_3$.

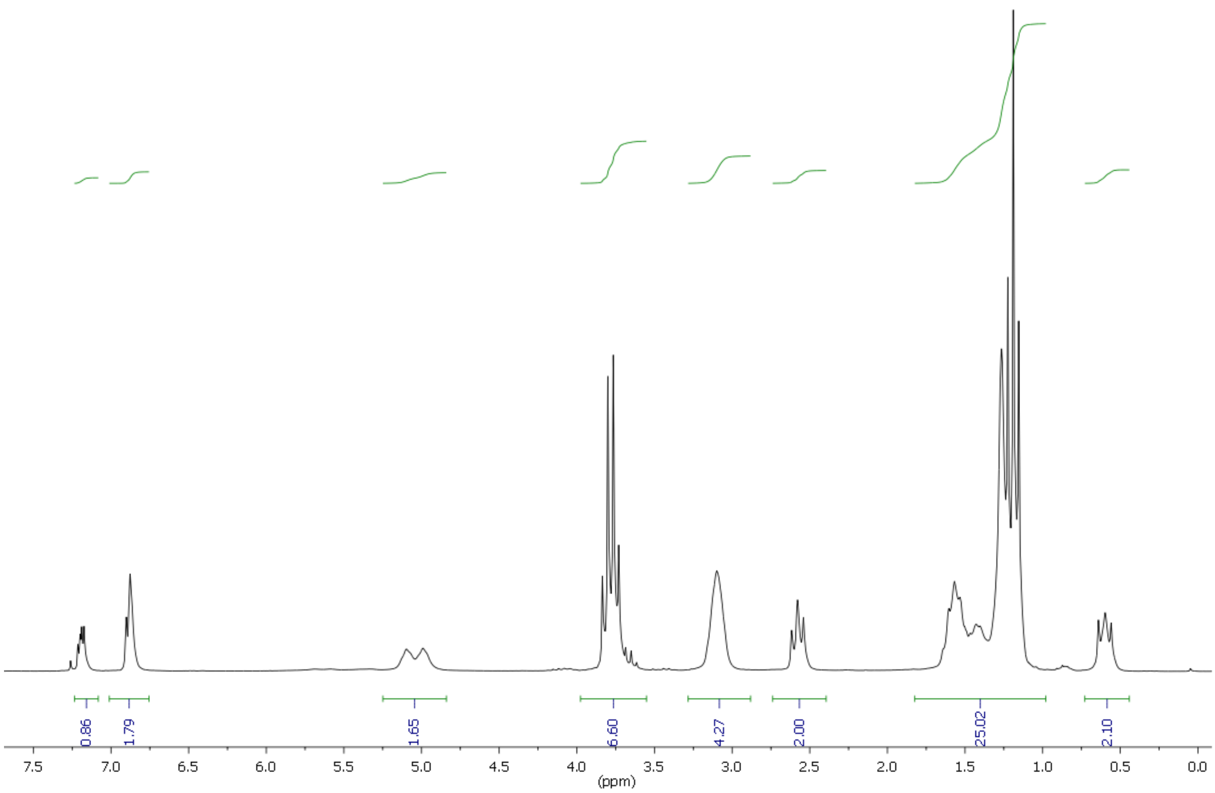

Figure S7 : ^{1}H NMR spectrum of **4** in $CDCl_3$.

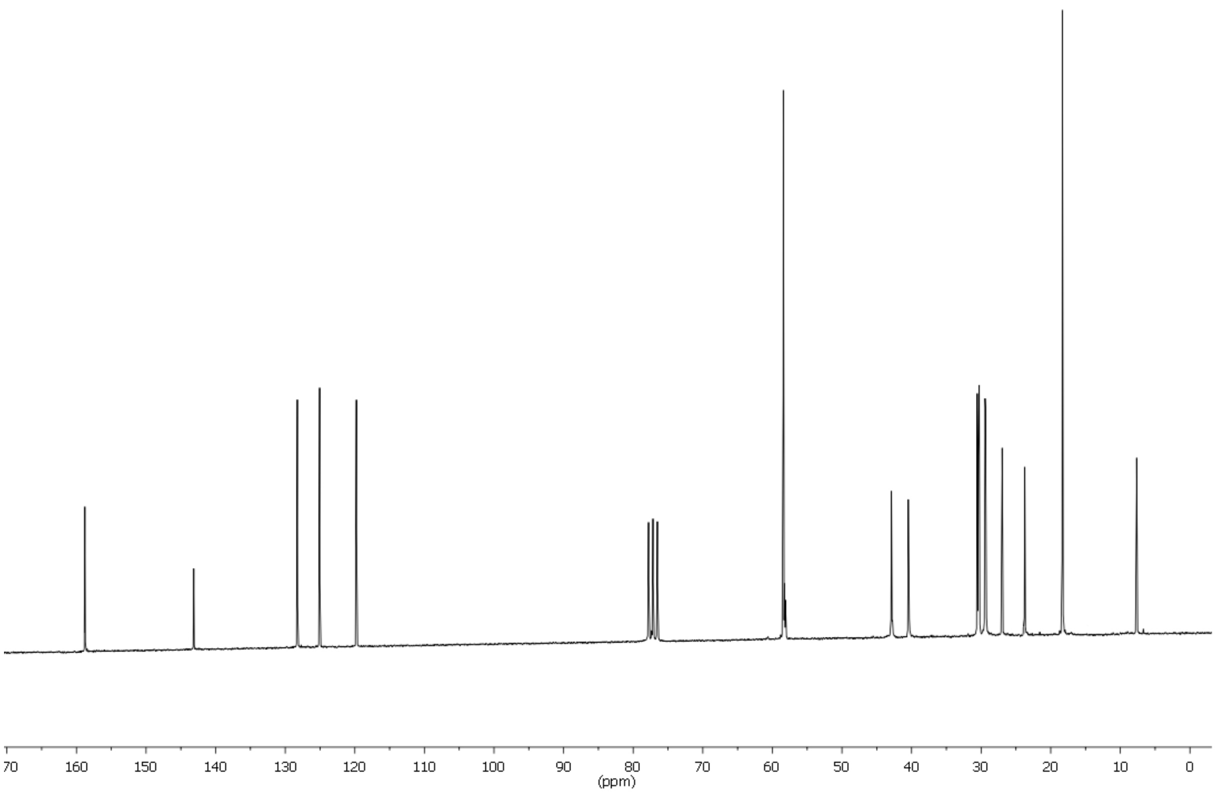

Figure S8 : $^{13}C\{^{1}H\}$ NMR spectrum of **4** in $CDCl_3$.

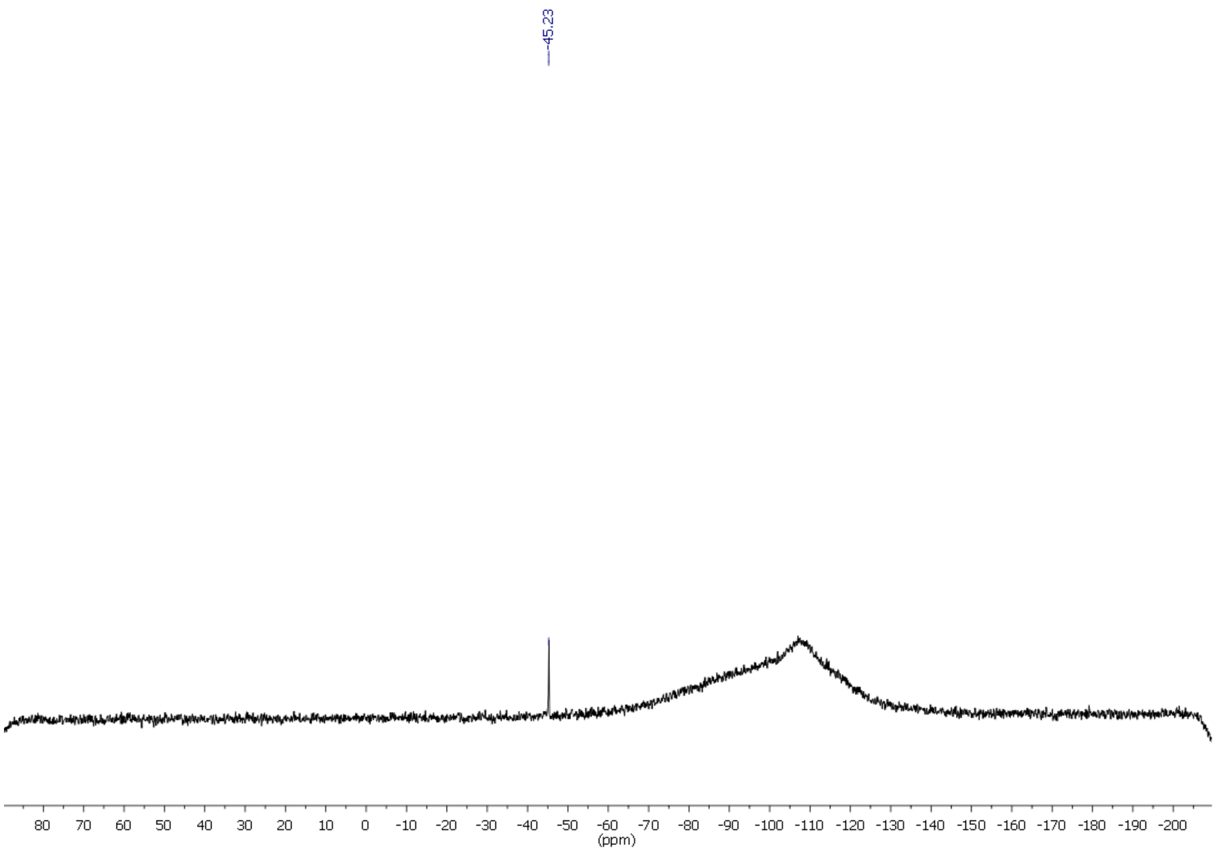

Figure S9 : $^{29}Si\{^{1}H\}$ NMR spectrum of **4** in $CDCl_3$.

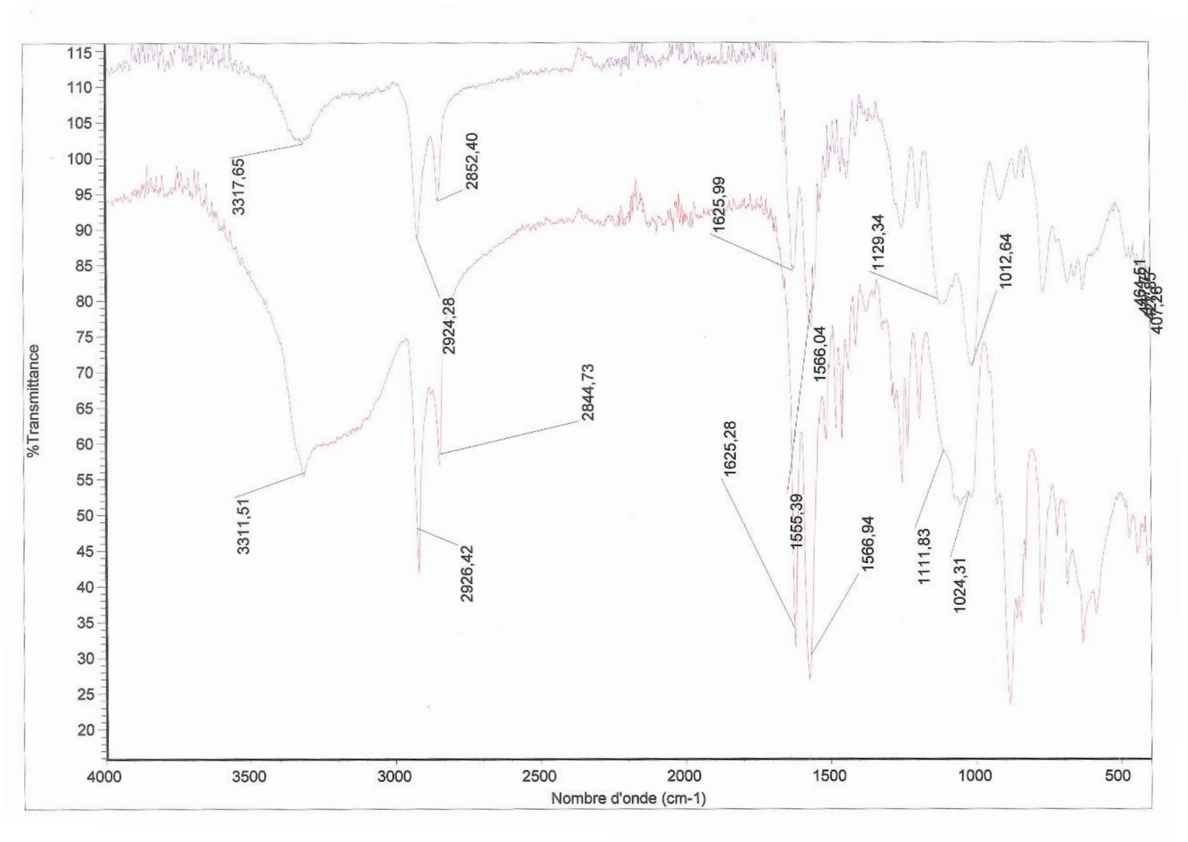

Figure S10: ATR-FT-IR spectra of hybrid materials $\mathbf{M4_{25}}$ (bottom) and $\mathbf{M4_{110}}$ (top).

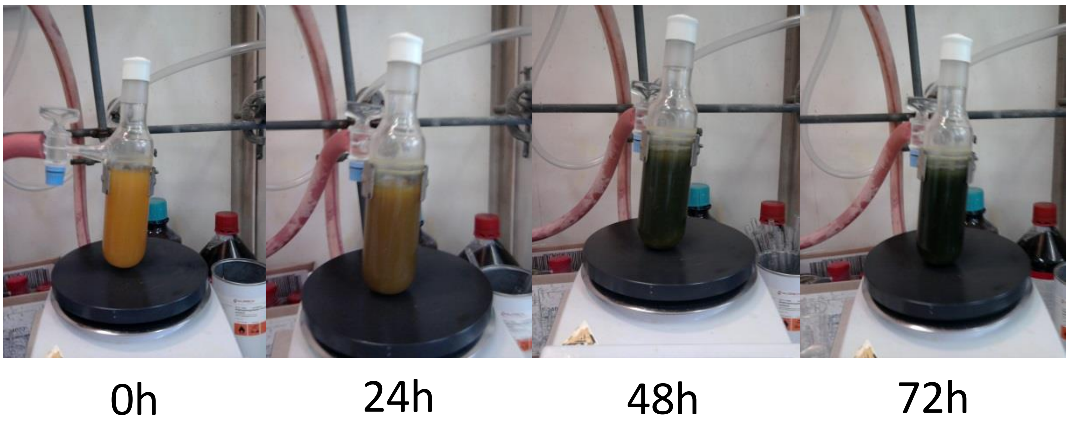

Figure S11: Colour evolution during the chemical polymerization of thiophene units in **$M4_{110}$**.

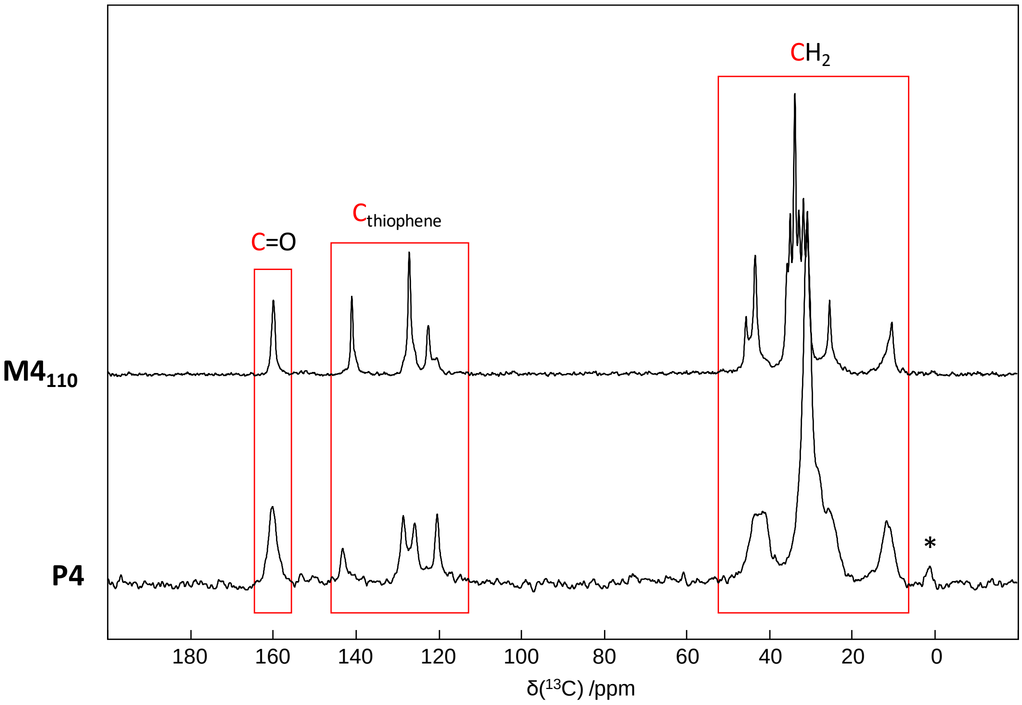

Figure S12: ^{13}C CPMAS solid state NMR spectra of **$M4_{110}$** and **P4**.

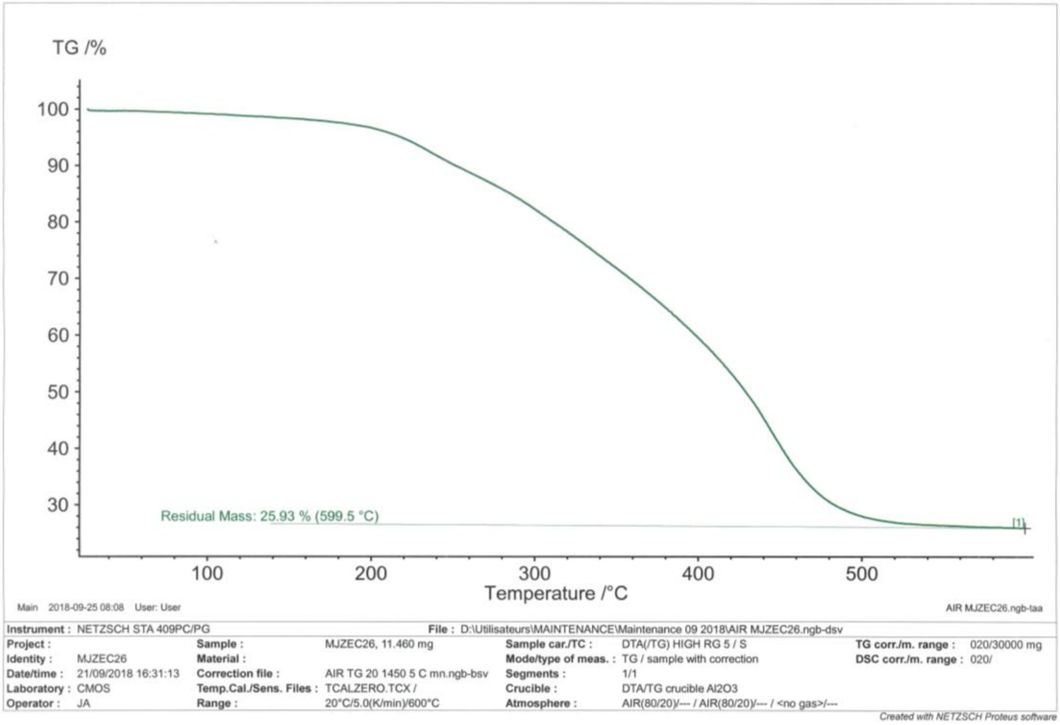

Figure S13: TGA curve of lamellar polythiophene-–silica hybrid material **P4**.
